# Hybrids as NO Donors

**DOI:** 10.3390/ijms22189788

**Published:** 2021-09-10

**Authors:** Ioanna-Chrysoula Tsopka, Dimitra Hadjipavlou-Litina

**Affiliations:** Department of Pharmaceutical Chemistry, School of Pharmacy, Faculty of Health Sciences, Aristotle University of Thessaloniki, 54124 Thessaloniki, Greece; joannatsopka@gmail.com

**Keywords:** cinnamic acid, NO donor, hybrids

## Abstract

Cinnamic acid and its derivatives have been studied for a variety of biological properties, including anti-inflammatory, antioxidant, anticancer, antihypertensive, and antibacterial. Many hybrids of cinnamic derivatives with other bioactive molecules have been synthesized and evaluated as nitric oxide (NO) donors. Since NO plays a significant role in various biological processes, including vasodilation, inflammation, and neurotransmission, NO donor groups are incorporated into the structures of already-known bioactive molecules to enhance their biological properties. In this review, we present cinnamic hybrids with NO-donating ability useful in the treatment of several diseases.

## 1. Introduction

In recent years, molecular hybridization has been extensively developed, focused on the preparation of novel hybrid drug candidates with improved pharmacokinetic profiles. These molecules can add to the treatment of diseases with complex etiology, in which the therapeutic approach usually involves a combination of cocktail drugs [1]. After long-term use, drugs acting on a specific biological target (single-target therapeutic agents) are likely to lead to the appearance of side effects, toxicity in the tissue in which they are based, and reduced effectiveness due to growing drug resistance. On the contrary, hybrid drug candidates are designed to act individually (each one separately on its own target), but also through a synergistic effect [2].

A number of hybrid NO-donor drugs have been developed through a molecular hybridization approach. The new hybrids retain the pharmacological activity of the parent compound, but they also act as NO donors.

In 1998, Furchgott, Ignarro, and Murad were awarded with the Nobel Prize for their discoveries, concerning nitric oxide (NO) as a signaling molecule in the cardiovascular system [3], where its role is the regulation of micro- and macrovascular homeostasis, the induction of vasodilation, and the inhibition of platelet aggregation [4]. Moreover, NO is an important factor in the nervous system, in a more complex way. It is a neurotransmitter at the end of noncholinergic and nonadrenergic nerves that control a variety of respiratory, gastrointestinal, and genitourinary functions [5]. In the immune system, it is one of the molecules that acts against pathogens of different origins, while it regulates T- and B-cell proliferation, leukocyte adhesion in the endothelium, production of cytokines, and growth inhibition of tumor cells [6].

Nitric oxide (NO) is a free radical involved in many biological processes. It is produced by a family of enzymes known as nitric oxide synthases (NOS), which catalyze the formation of L-arginine to L-citrulline. There are three different isoforms in mammalian organisms: endothelial nitric oxide synthase (eNOS), which is mainly expressed by endothelial cells; neuronal nitric oxide synthase (nNOS), which is expressed in central and peripheral neurons; and the inducible isoform (iNOS), which is expressed in a variety of cell types under both normal and pathological conditions, including macrophages, vascular endothelial, and epithelial cells [7]. Expression of iNOS occurs in conditions of inflammation, and produces large amounts of NO [8,9].

In the presence of oxygen, NO is rapidly converted to peroxynitrite (OONO^−^), a very reactive molecule capable of splitting into secondary radicals (OH^-^ and NO_2_^−^). These free radicals can induce activation of lipoperoxidation processes, oxidation of sulfhydryl groups of proteins, changes of valence of metal ions, and an increase of nitrosamines content that cause functional disorders in the cellular membranes and intracellular proteins [10].

Eicosanoids produced by cyclooxygenases (COXs) and lipoxygenases (LOXs) can reduce iNOS expression and NO production, while stimuli that enhance iNOS and NO formation also may induce COX-2 expression. In numerous reports, NO donors have been reported to stimulate or inhibit prostaglandin biosynthesis in a variety of cellular systems. It is likely that NO does not mediate all these actions, but NO-derived species such as peroxynitrite or nitrosothiols may be responsible [11].

**Figure 1:**
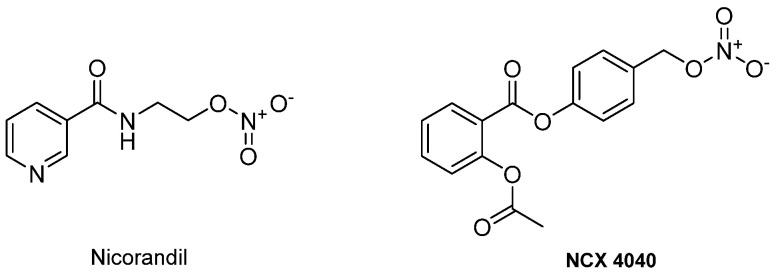
Nicorandil and the derivative NCX 4040.

Since the discovery of NO in early 1980s, many molecules such as furoxans, nitrates, and S-nitrosothiols have been evaluated for their capability to release NO (NO donors) [12]. In the last decade, many hybrid compounds containing a NO donor group have been synthesized to treat a wide range of diseases. These hybrid compounds are examples of multitarget drugs, the single chemical entities of which can simultaneously modulate more than one target, such as nicorandil and the derivative NCX 4040 (Figure 1). Nicorandil has a dual activity as an organic nitrite and as an ATP-dependent potassium channel agonist, and can be used in the treatment of angina pectoris [13]. The structure of NCX 4040 combines salicylic acid and a NO donor. In in vitro and in vivo experiments, it was shown that the presence of a NO donor was necessary for the anticancer activity of the hybrid [14].

Cinnamic acid is a natural product that it is found in various essential oils, resins, and balsams. In biological chemistry, is a key intermediate in the shikimate and phenylpropanoid pathways, being a precursor of the flavonoids and the plant structural component lignin. It belongs to the class of auxin, which is recognized as plant hormone regulating cell growth and differentiation [15,16].

The four most common hydroxy-substituted cinnamic acids in nature are *p*-coumaric, caffeic, ferulic, and sinapic acids, while *o-* and *m-* coumaric acid are less frequent. These compounds do not usually exist in free form, unless they are products of chemical or enzymatic hydrolysis [17].

**Figure 2:**
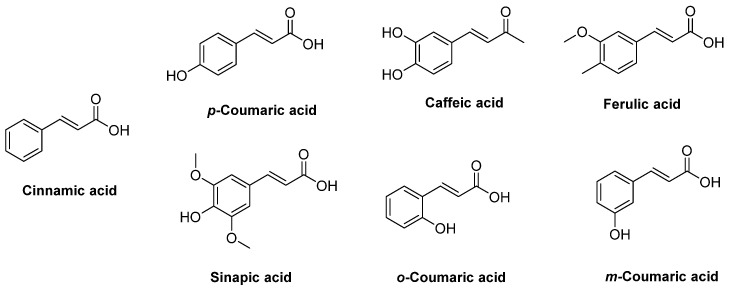
Chemical strustures of cinnamic acid and its derivativies.

Cinnamic acid and its derivatives (Figure 2) present a variety of biological activities, such as antioxidant [18], antimicrobial [18], anticancer [19], anti-inflammatory [20], antidiabetic [21], hepatoprotective [22], antimalarial [23], antituberculosis [24] and antifungal [25]. However, the combination of appropriate pharmacophore groups with suitable substituted cinnamic acids can lead to conjugates with anti-inflammatory activities.

Since cinnamic acid derivatives show a variety of biological activities, many cinnamic acid hybrids have been synthesized and tested as NO donors [14].

The aim of this review is mainly to describe the chemical structures of the hybrid molecules combining the cinnamic acid pharmacophore and the nitric oxide donor (NO donor) group, to explore the latest findings of this strategy, and to present the main biological activities and applications of these molecules. This information will be helpful for scientists working in this scientific field. Recently, a member of our group presented the review “Cinnamate Hybrids: A Unique Family of Compounds with Multiple Biological Activities”, covering in general all the published research on cinnamate hybrids [26]. This publication listed the biological activities and applications of several cinnamate hybrids. However, it did not describe the case of cinnamate hybrids as NO donors. Thus, we found it interesting to look deeper into the recent literature and find appropriate examples of cinnamic hybrids acting as NO donors, and to discuss their biological applications.

The content list includes, depending on the structure and the activities, the following:

- Nitro-oxy esters of cinnamic acids
  (a) *Nitro-oxy ester hybrids as anti-inflammatory and antioxidant agents;*
  (b) *Nitro-oxy ester hybrids as anti-atherosclerotic agents;*
  (c) *Nitro-oxy ester hybrids as anticancer agents;*
  (d) *Nitro-oxy ester hybrids as multifunctional acetyl- and butyrylcholinesterase inhibitors;*
  (e) *Nitro-oxy ester hybrids as antiplatelet and antithrombotic agents.*
- Furoxan-cinnamic acid hybrids
  (a) *Furoxan hybrids as anti-atherosclerotic agents;*
  (b) *Furoxan hybrids as anticancer agents;*
  (c) *Furoxan hybrids as anti-diabetic agents.*

## 2. Nitro-Oxy Esters of Cinnamic Acids

### 2.1. Nitro-Oxy Ester Hybrids as Anti-Inflammatory and Antioxidant Agents

Inflammation is a normal response of the body in cases of histological lesion, and is divided into acute and chronic. Chronic inflammation can be present in a variety of multifactorial diseases such as cancer, diabetes, arthritis, Alzheimer’s, and autoimmune and cardiovascular diseases [27].

COX and LOX are important enzymes involved in the onset of inflammation [28,29]. Many well-known nonsteroidal anti-inflammatory drugs (NSAIDs) are aimed at inhibiting COX, while LOX has been the target of a series of cinnamic acids [30,31]. In recent years, researchers have reported that the incorporation of NO donors in NSAIDs can reduce the side effect of gastric ulcer, and may have a potential synergistic activity. In addition, the interaction of NO and superoxide anions can modulate the inflammation process, interfering in COX function and cytokine synthesis [32].

Oxidative stress is related to inflammation. Under normal conditions, reactive oxygen species and endogenous antioxidant defense mechanisms are in balance. However, an imbalance can lead to oxidative stress that causes numerous diseases, and is associated with inflammation diseases [33].

**Figure 3:**
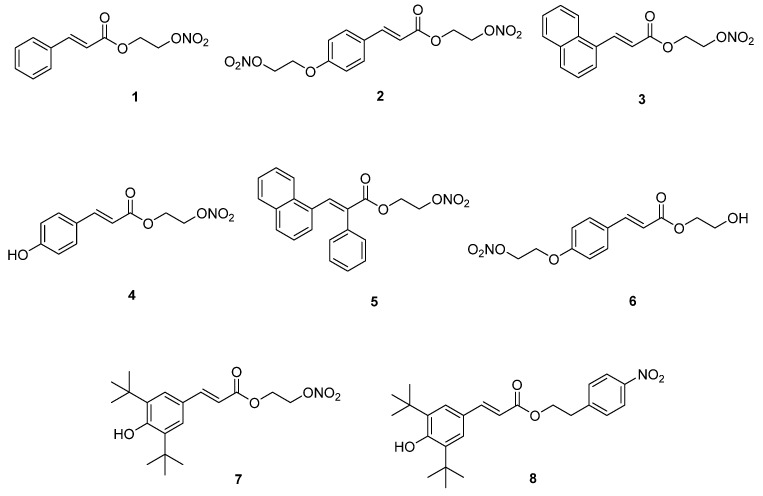
Chemical structure of the nitrooxy esters of cinnamic acids **1**–**8** with anti-inflammatory and antioxidant activities [33].

A new series of synthetic cinnamic acid derivatives (Figure 3) incorporating a nitro group were evaluated as anti-inflammatory and antioxidant agents by Fotopoulos et al. [34]. The new molecules tested as possible inhibitors of soybean LOX and ovine COX–2. Hybrids **1**–**6** did not present high or any LOX inhibition at 100 μM, with compound **1** as the most potent LOX inhibitor of the series with 41% inhibition at 100 μM, while the control Nordihydroguaiaretic acid (NDGA) showed a 93% inhibition at 100 μM. Compound **2** displayed an inhibition activity of COX with a value of 62.5% at 100 μM, in comparison to indomethacin (95% inhibition at 100 μM). This particular compound showed 55.5% inhibition of carrageenin edema in vivo. The in vivo activity of **2** was higher than indomethacin (37.3%), followed by the compounds **1**, **3**, and **4**, which presented 24–36% inhibition.

In addition, hybrids **1**–**6** were tested for their in vitro antioxidant activity. Hybrids **1**, **3**, **5**, and **6** showed the highest antilipid peroxidation activity, in a range of 67.4–86.5% at 100 μM. All hybrids were found to be less potent than trolox (88%). Lipophilicity was found to influence antioxidant activity.

Hyperlipidemia is a medical term for abnormally high levels of lipids or lipoproteins in the blood. In recent years, research studies indicated an association between hyperlipidemia, inflammation, and ROS production. Nobelos et al. [35] reported the anti-dyslipidemic activity of the synthesized cinnamic hybrids **1** and **7** (Figure 3), which were evaluated in Triton-induced hyperlipidemia in rats. Hybrid **1** showed 54.4–74.8% reduction of total cholesterol, triglycerides, and LDL-cholesterol. The results were comparable to simvastatin (70.0–73.0%), and better than ibuprofen (38.0–41.6%) and naproxen (25.5–53.0%). An increase in values of reduction of lipidemic indices also was demonstrated by hybrid **7** (45.7–65.5%).

Hybrids **7** and **8** displayed high in vitro and in vivo anti-inflammatory activity. The in vitro evaluation of these hybrids against soybean lipoxygenase 1-B, using linoleic acid as a substrate, presented considerable activity, with IC_50_ values of 44 μΜ and 10.5 μM, respectively. The hybrids were more potent than the standard reference drugs ibuprofen (200 μM), ketoprofen (220 μM), and trolox (>300 μM), but not nordihydroguaiaretic acid (NDGA, 1.3 μM). The in vivo anti-inflammatory effect of the hybrids was tested on a carrageenin-induced paw edema model. The new molecules showed in vivo inhibition of inflammation, 55% and 51%, respectively, and were more potent than the classic NSAIDs drugs ibuprofen (36%), indomethacin (42%), naproxen (11%), and ketoprofen (47%). The results indicated that the higher potency of compound **8** was correlated to higher lipophilicity.

Furthermore, the hybrids presented in vitro antioxidant activity, scavenging the DPPH radical, and inhibition of lipid peroxidation. Hybrids **7** (21% interaction at 50 μM) and **8** (23% interaction at 50 μM) were not as potent scavengers as Trolox (38% at 50 μM). Hybrid **7** was found to be a potent inhibitor of lipid peroxidation, with IC_50_ of 41 μM (trolox 25 μM), while hybrid **8** (IC_50_ = 150 μM) did not show a considerable activity.

Moreover, these compounds were tested for their capability to release NO in vitro, using the Griess reagent test. Hybrids **1** and **7** did not present higher activity than S-nitroso-*N*-acetylpenicillamine (SNAP), a known NO donor with a measured activity of 56.3 μM at 100 μM.

### 2.2. Nitro-Oxy Ester Hybrids as Antiatherosclerotic Agents

Atherosclerosis is a main cause of cardiovascular diseases. Increased levels and oxidative modification of low-density lipoprotein (LDL), as well the dysfunction of NO-mediated pathways that lead to low concentration levels of NO, are significant risk factors. Research studies showed that antioxidant agents are capable of decreasing the impact of atherosclerosis through a regulation mechanism [36,37].

**Figure 4:**
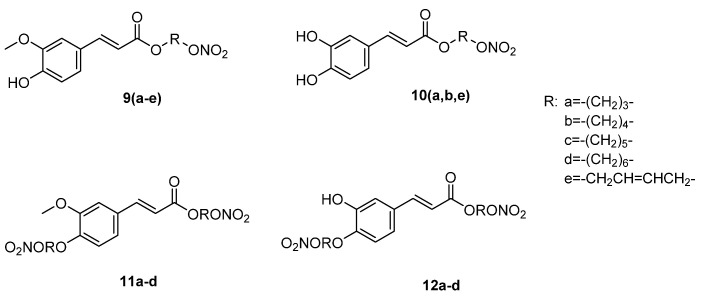
Chemical structure of the nitrooxy esters of cinnamic acids **9**–**12** with anti-atherosclerotic and anticancer activities [36,37].

Nian-Guang Li et al. [37,38] synthesized two series of hybrid molecules (Figure 4) as potential antiatherosclerotic drug candidates. In particular, ferulic and caffeic acids combined with a nitro group through various linkers. In both series, antioxidant activity was measured using the DPPH and lipid peroxidation assays. Within the series of hybrids **9**, none presented antioxidant activity higher than ferulic acid. Hybrid **9a** was found to be the most potent, with IC_50_ values of 31.3 μM (DPPH) and 39.1 μM (lipid peroxidation), followed by hybrids **9b** (54.9 μM at DPPH, 56.9 μM at lipid peroxidation) and **9c** (69.3 μM at DPPH, 67.2 μM at lipid peroxidation). The results indicated that an increased number of carbon atoms decreased the antioxidant activity. In addition, caffeic hybrids **10a** (0.011 mM at DPPH, 0.022 mM at lipid peroxidation) and **10e** (0.012 mM at DPPH, 0.027 mM at lipid peroxidation) were as potent as caffeic acid (0.009 mM at DPPH) in DPPH-scavenging assays. However, hybrid **10b** (0.031 mM at DPPH, 0.048 mM at lipid peroxidation) showed lower activity compared to the control caffeic acid in DPPH assays.

Free phenolic hydroxyl groups of cinnamic acids are considered to be important for the presence of antioxidant activity. However, compounds **11** and **12** did not show a good antioxidant profile. In particular, caffeic acid derivatives **12**, with one substituted phenolic hydroxyl group, seem to be less potent than hybrids **10**, with IC_50_ values in a range of 0.67–0.97 mM (DPPH and lipid peroxidation assays). In addition, no activity was recorded for ferulic acid hybrids **11**, since both phenolic hydroxyl groups of the molecules are occupied.

The vasodilating activity of the hybrids was measured through an in vitro vascular relaxation assay (organ bath) using PGF2a-precontracted porcine pulmonary arteries. Dinitric ferulic hybrids **11** showed a considerable activity, with EC_50_ values of 0.5231–0.7267 μM, while ferulic mononitric hybrids **9** were less potent (EC_50_ of 12.67–16.85 μM). In addition, caffeic dinitric hybrids **12** (EC_50_ = 1.89–3.39 μM) were 2- to 3-fold more active than mononitric esters **10** (EC_50_ = 4.32–6.17 μM). None of the hybrids **9**–**12** had higher activity than isosorbide dinitrate (ISDN), with EC_50_ of 0.11 μM.

The hybrids were evaluated in vitro as NO donors through a Griess test. Mononitrates of ferulic and caffeic acid **9** and **10** generated a minimum amount of NO (1.00–4.42 μM at 2 h), while dinitrates **11** and **12** (8.06–12.10 μM at 2 h) showed an NO ability, comparable to ISDN (12.14 μM at 2 h). However, a measurement at 2 h and 4 h indicated that the NO release time of dinitrates **11** and **12** was much shorter than that of corresponding mononitrates.

### 2.3. Nitro-Oxy Ester Hybrids as Anticancer Agents

Drug resistance is the major problem of induced effectiveness of most chemotherapeutic agents, leading to the development of new molecules [39]. Antioxidant moieties are often used in hybrid molecules to carry potential anticancer activity. They were described to have damaged DNA and other cellular molecules [40].

Since cinnamic acids are potent antioxidants and were found to present satisfactory anticancer activities in a variety of cancer types, they have been used as a useful scaffold for the development of improved antitumor drugs [19]. Moreover, it seems that NO is capable of killing a variety of tumor cells directly or indirectly. However, the precise mechanism of NO in cancer remains unknown [41].

Research on hybrids **9**, **10**, **11**, and **12**, tested in vitro in 14 cancer cell lines, was published in 2012. The unsaturated hybrid nitrates **9e** and **10e** were more potent than the saturated ones **9a**–**d** and **10a,b**, with IC_50_ values less than 10 μM. In addition, IC_50_ values of caffeic hybrid **10e**, in comparison to ferulic analogue **9e**, underlined the importance of the phenolic hydroxyl groups to enhance the anticancer activity. Hybrids **11** and **12**, with dual nitrate groups containing one or no free phenolic hydroxyl groups, did not present any activity in the majority of the tested cancer cell lines [42]. All the IC_50_ values are given in Table 1 and Table 2.

### 2.4. Nitro-Oxy Ester Hybrids as Multifunctional Acetyl- and Butyrylcholinesterase Inhibitors

Alzheimer’s disease is a progressive neurodegenerative disorder. According to the cholinergic hypothesis of the pathogenesis of the disease, dysfunction of the cholinergic system, and mainly, decreased levels of acetylcholine (ACh), lead to cognitive and memory deficits. However, the activity of acetylcholinesterase (AChE) and butyrylcholinesterase (BuChE) can modulate the ACh levels [43,44].

**Figure 5:**
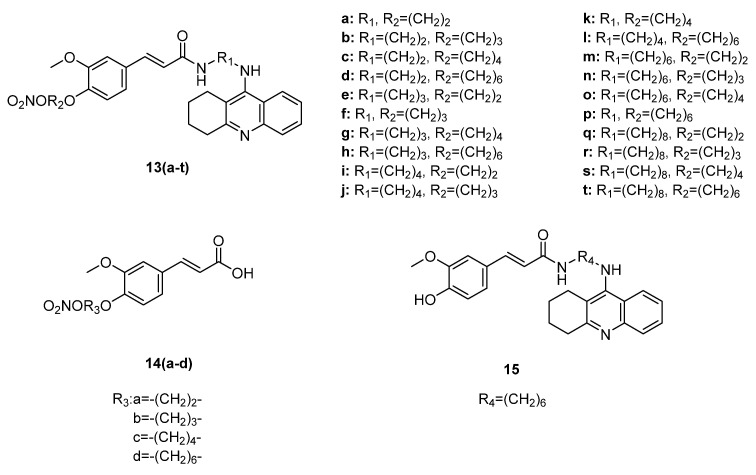
Chemical structure of cinnamic hybrids **13**, **14** and **15** as acetyl- and butyrylcholinesterase inhibitors [44].

Yao Chen et al. [45] reported hybrids of tacrine, an AChE inhibitor, combined with ferulic acid and a nitro group, as potential multifactorial acetyl- and butyrylcholinesterase inhibitors. The new molecules (Figure 5) were examined for their ability to inhibit AChE from *Electrophorus electricus* (eeAChE) and BuChE from equine serum, following Ellman’s method. All hybrids were more potent inhibitors of AChE than tacrine (IC_50_ = 69.8 nM). Increased activity was shown by hybrids **13n**–**q** (IC_50_ = 3.7–5.5 nM). Hybrid **13g**, with an IC_50_ value of 3.6 nM, was the most potent. Hybrids **13o**–**13t** presented the best BuChE inhibitory activity (IC_50_ = 1.0−2.0 nM), with **13r** as the most potent inhibitor. Most of the new molecules exhibited higher (IC_50_ = 10.6 nM) inhibitory activity of BuChE than tacrine. Furthermore, nitro-ferulic hybrids **14** were tested for their ability to inhibit AChE and BuChE, but none of them showed any activity.

The new hybrids **13a**–**t** were tested for their antioxidant activity, but none of them showed more than 1% free-radical-scavenging activity in the DPPH assay. Hybrid **15**, without a NO donor group and having a free phenolic hydroxy group, presented a 64.7% free-radical-scavenging activity.

All the compounds showed increased levels of nitrite in comparison to tacrine, which was used as a negative control. Hybrids **13f** and **13q** appeared to be the most potent, with values of 0.314 μg/mL and 0.301 μg/mL for nitrite, respectively. These values were found close to the value of the positive control, isosorbide dinitrate (ISDN, 0.382 μg/mL).

The vasodilation activities of hybrids **13a**, **13f**, and **13q** were evaluated in an ex vivo organ bath (coronary arteries from rat). None of them was more potent than ISDN (EC_50_ = 25.2 μM), whereas hybrid **13f** showed a considerable activity, with an IC_50_ value of 34.3 μM.

### 2.5. Nitro-Oxy Ester Hybrids as Antiplatelet and Antithrombotic Agents

Thromboembolic diseases such as myocardial infarction, ischemic stroke, acute atherosclerosis, and pulmonary embolism are caused by the formation of blood clots in blood vessels. NBP (3-*n*-butylphtalide) was approved by the State Food and Drug Administration (SFDA) of China as a drug for the treatment of ischemic stroke.

**Figure 6:**
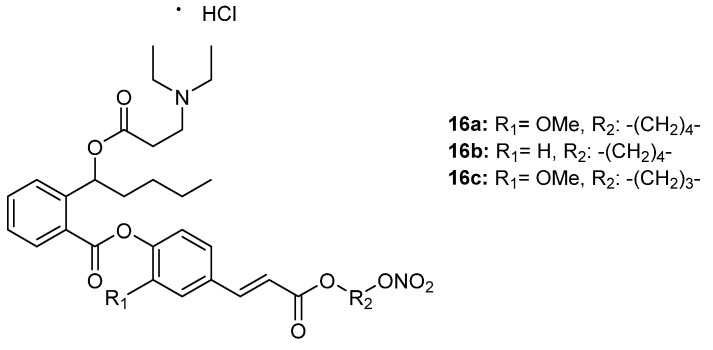
Chemical structure of cinnamic hybrids **16** with antiplatelet and antithrombotic activities [45].

As NBP presented a poor pharmacochemical profile, Wang et al. [46] synthesized NBP–NO donor hybrids (Figure 6). Ferulic and *p*-hydroxyl cinnamic acid were used as linkers, since both possess antioxidant and antiplatelet aggregation activities. The in vitro antiplatelet properties of the new compounds were tested in rabbit platelet-rich plasma (PRP) using Born’s turbidimetric method. The most potent was **16a**, with 88.5% inhibition of platelet aggregation, following by **16b** and **16c**, with 70.5% and 60.6% inhibition, respectively. None of them presented better activity than aspirin (73.7%); however, they were more potent than NBP (51.4%).

Correlation between NO release and antiplatelet aggregation activity was detected. In particular, hybrids **16a**, **16b**, and **16c** showed the best values for NO, releasing levels of 0.3, 0.28, and 0.2 μg/mL, respectively.

Hybrids **16a** and **16b** proceeded to further in vivo studies for antithrombotic activities, specifically on the formation of thrombus in a rat extracorporeal circulation of an arteriovenous (A-V) cannula model. Only hybrid **16a** showed higher activity than NBP (29.97 mg) and aspirin (30.73 mg). The thrombus weight of **16a**-treated rats was found to be 27.95 mg.

## 3. Furoxan–Cinnamic Acid Hybrids

### 3.1. Furoxan Hybrids as Antiatherosclerotic Agents

**Figure 7:**
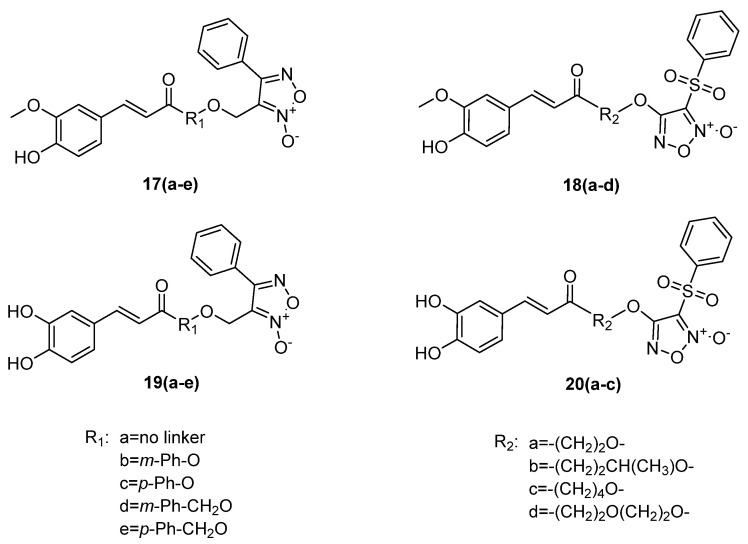
Chemical structure of the furoxan–cinnamic acid hybrids **17**–**20** with anti-atherosclerotic and anticancer activities [36,37].

Nian-Guang Li et al. [37,38] synthesized a series of furoxan–cinnamic acid hybrids as potential antiatherosclerotic drug candidates. Ferulic acid hybrids **17** and **18** (Figure 7), as well as monoesters **9a**–**c** (Figure 5), did not present better antioxidant activity than ferulic acid (IC_50_ = 16.2 μM for DPPH, IC_50_ = 17.6 μM for lipid peroxidation). Hybrid **18c**, the most potent antioxidant, showed the same activity as **9c**, with IC_50_ values of 66.9 μM (DPPH) and 72.1 μM (lipid peroxidation).

Caffeic hybrids **19** and **20** (Figure 7) presented a higher antioxidant activity (IC_50_ = 13–39 μM in DPPH assay, IC_50_ = 25–56 μM in lipid peroxidation assay) compared to ferulic hybrids **17** and **18**. The phenolic hydroxy group seems to be significant for the antioxidant activity of the molecules. The most potent antioxidant agent was found to be hybrid **19a**, with an IC_50_ value of 13 μM in DPPH and 25 μM in lipid peroxidation. This hybrid combines a phelylfuroxan moiety and caffeic acid with no linker. In general, phenylfuroxan hybrids **19** had better antioxidant activity than phenylsulfonylfuroxan hybrids **20** [38].

The vasodilating activity of the hybrids was measured through an in vitro vascular relaxation assay (organ bath) using PGF2a-precontracted porcine pulmonary arteries. The most active compounds belonged to the phenylsulfonylfuroxan series **18**. In particular, hybrid **18c**, with a higher antioxidant activity among the furoxan hybrids **17** and **18**, was more potent than ISDN (EC_50_ = 0.1123 μM), with an EC_50_ value of 0.0928 μM, followed by hybrids **18b** and **18d** (which were as potent as ISDN), with EC_50_ values of 0.1143 μM and 0.1039 μM, respectively [37]. Phenylfuroxans of caffeic acid **19** demonstrated lower EC_50_ values compared to the corresponding phenylsulfonylfuroxans **20** (EC_50_ = 1.03–1.83 μM). The best vasodilating activity was shown by hybrid **19a** (EC_50_ = 0.12 μM), the molecule with the higher antioxidant activity [38].

Hybrids **17** exhibited higher NO release potency (3.71–6.58 μM at 2 h) than mononitrates **9**, but not as good as dinitro hybrids **12**, with the exception of **17a**, with a value of 10.70 μM at 2 h. Correlation observed between vasodilating activities and NO-releasing potency of the new ferulic molecules; e.g., phenylsulfonyl hybrids **18** were the most potent NO donors, with values of 22.06–27.53 μM at 2 h. In addition, caffeic hybrids **19**, with the best vasodilating activities in comparison to hybrids **20**, presented NO release in a range of 9.05–11.08 μM, and were as potent as ISDN. Phenylsulfonylfuroxans of caffeic acid values released were found from 2.29 to 3.82 μΜ.

### 3.2. Furoxan Hybrids as Anticancer Agents

Li et al. [42] tested furoxan hybrids **17**, **18**, and **19a** in 14 cancer cell lines. The most promising anticancer agents seemed to be compounds **18b**–**d**, presenting decreased IC_50_ values (0.40–2.88 μM) against all the human cancer cells (Table 3 and Table 4). The results indicated that the linkers played an important role in the appearance of anticancer acti-vity. Caffeic hybrid **19a** showed better values than ferulic hybrids **17**, with the exception of the corresponding hybrid **17a**.

**Figure 8:**
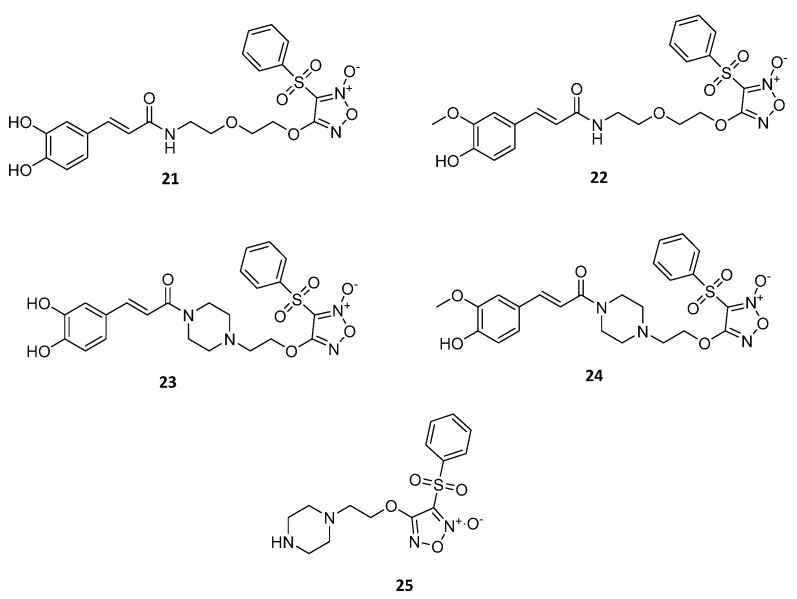
Chemical structure of the of furoxan–cinnamic acid hybrids **21**–**24** and furoxan moiety **25** with anticancer activity [46].

In another research study, Ming-Dong Lu et al. [47] synthesized NO-releasing derivatives of cinnamic acids (Figure 8) as antitumor agents. The hybrid compounds consisted of one furoxan moiety and a hydroxylcinnamic acid derivative, coupled with various alkyl amines as linkers. The new molecules were evaluated for their in vitro antitumor activity against hepatocellular carcinoma cells (SMMC-7721 and HepG2) and human breast cancer cells (MCF-7). As a result, none of the tested hybrids were a more potent inhibitor than Adriamycin in all three cancer cell lines; however, hybrid **21** demonstrated the best inhibitory activity, with IC_50_ values of 6.1 μM (SMMC-7721), 7.3 μM (HepG2), and 3.8 μM (MCF-7). This hybrid proceeded to further studies and presented a selective cytotoxic effect. Specifically, it did not present important inhibition potency in nontumor cells (liver LO2 cells), but a high inhibition effect of HepG2 cell proliferation (2–16 μM), in a concentration-dependent manner. Hybrid **22**, the ferulic analogue of hybrid **21**, also displayed a significant antitumor activity, with IC_50_ values of 7.9 μM (SMMC-7721), 8.2 μM (HepG2), and 6.5 μM (MCF-7). Moreover, hybrids **23** and **24** were found to be more potent than the corresponding NO donor moiety **25**, with IC_50_ values of 5.0–10.8 μM, as shown in Table 5, suggesting the contribution of the furoxan moiety to the antitumor activity of hydroxycinnamic acids.

### 3.3. Furoxan Hybrids as Antidiabetic Agents

Metabolic syndrome is a cluster of conditions occurring together, including increased blood pressure, high blood sugar, abnormal cholesterol and triglyceride levels, raised fasting glucose, and central obesity. This disorder can lead to the development of cardiovascular disease (CVD), renal and liver diseases, and type 2 diabetes mellitus (T2DM). The systemic inflammatory process, associated with the metabolic syndrome, presents numerous deleterious effects that promote plaque activation and the appearance of atherosclerosis. It has been reported that antioxidants and radical scavengers prevent the formation of advanced glycation end-products (AGEs) formed after long sustained hyperglycemia [48,49].

**Figure 9:**
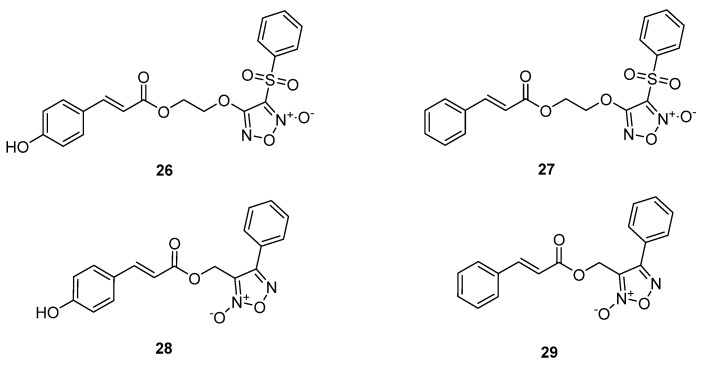
Chemical structure of furoxan–cinnamic acid hybrids **26**–**29** as antidiabetic agents.

Xie et al. combined cinnamic acids with NO donor groups as a therapeutic approach against metabolic syndrome [50] and diabetes mellitus [51]. In particular, phenylsulfonyl- and phenylfuroxan alcohols were linked with some natural cinnamic acid derivatives (Figure 7 and Figure 9). In vitro evaluation of phenylsulfonyl hybrids **18a**, **20a**, **26**, and **27** against yeast a-glucosidase from Saccharomyces cerevisiae showed that hybrid **20a** (IC_50_ = 187.34 μM) was more potent than acarbose (IC_50_ = 232.41 μM). Hybrids **18a** and **26** were comparable to acarbose, with IC_50_ values of 263.47 μM and 278.54 μM, respectively, while hybrid **27** presented an increased IC_50_ value [50].

In vitro evaluation of phenylfuroxan hybrids **17a**, **19a**, **28**, and **29** against a-glucosidase (maltase and sucrase) from rat small intestine showed that only hybrids **17a** (IC_50_ = 301.73 μM in maltase and 223.36 μM in sucrase) and **19a** (IC_50_ = 123.36 μM in maltase and 155.03 μM in sucrase) exhibited any activity. None of the hybrids presented better inhibitory activity than acarbose (5.59 μM in maltase and 2.31 μM in sucrase), and hybrids **28** and **29** did not show any activity [51].

The antiglycosylation activity of the compounds was determined in a bovine serum albumin (BSA)-methylglyoxal (MGO) system, which simulated the glycation reactions of the body. The inhibition of AGEs formation was increased by the hybrids **18a** and **20a** (IC_50_ values of 0.985 and 0.158 mM, respectively), which presented higher activity than aminoguanidine (IC_50_ = 1.510 mM). Phenylfuroxans **17a** and **19a** were not as potent as the control aminoguanidine, with IC_50_ values of 3.259 mM and 3.037 mM, respectively. Moreover, hybrids **26**, **27**, **28**, and **29** did not show any activity [50,51].

The in vitro antioxidant activity of the hybrids was tested using vitamin C as standard. DPPH and OH radical assays were used. Hybrid **20a** was found to be more potent than vitamin C, with IC_50_ of 0.042 mM (DPPH), 0.219 mM (OH radical), and 0.221 μM (anti-lipid peroxidation). Hybrid **18a** displayed antioxidant activity comparable to vitamin C, with IC_50_ of 0.181 mM (DPPH) and 0.669 mM (OH radical) and showed to be more potent than the control in antilipid peroxidation, with IC_50_ of 5.132 μM. However, hybrids **26** and **27** did not present good or any activity, suggesting that the absence of phenolic hydroxyl groups decreased the activity [50]. In the DPPH and OH radical assays, hybrid **19a**, containing two free phenolic hydroxyl groups, was determined to be the more potent antioxidant compared to ascorbic acid, with an IC_50_ of 0.0082 mM (DPPH) and 0.222 mM (OH radical). Hybrid **17a** showed an IC_50_ of 0.177 mM (DPPH) and 0.895 mM (OH radical), comparable to ascorbic acid activity. Removal of the methoxy group led to a reduced activity, since hybrid **28** showed an IC_50_ value of 0.987 mM (DPPH) and no activity in the OH radical assay. Hybrid **29** did not present any activity in either assay [51]. The results supported that hybrids with antioxidant activity have good antiglycation properties. Moreover, combination of these properties is more efficient in inhibiting the glycation reactions.

The antiplatelet aggregation activity of the hybrids was determined using adenosine diphosphate (ADP)-induced platelet aggregation in human platelet-rich plasma (PRP) through Born’s turbidimetric method. All phenylsulfonylfuroxan compounds were more potent than aspirin (5.96% inhibition at 1.5 μΜ), used as control. Hybrid **20a** showed the highest inhibition of 73.54% at 1.5 μM, followed by hybrids **18a**, **26**, and **27**, with an inhibition of 65.53%, 58.14%, and 56.44% at 1.5 μM, respectively [50]. Phenylfuroxans **17a**, **19a**, **28**, and **29** did not present higher activity than aspirin (28.11% at 0.15 mM), with 9.46%, 17.66%, 10.78%, and 12.66% inhibition at 0.15 mM, respectively [51].

The NO-releasing activity of the new compounds was correlated to the antiplatelet aggregation activity. The potency of the hybrids to release NO was estimated using a Griess reagent assay. Compound **20a** released a considerable amount of NO (45.01 μM), followed by hybrids **27**, **18a**, and **26** (40.01, 39.20, and 35.72 μM respectively). The results showed that phenolic hydroxyl may enhance the NO-release activity of the compounds [50], whereas the presence of the sulfonyl group may enhance the antiplatelet aggregation activity of the compounds. Phenylfuroxan hybrids **17a**, **19a**, **28**, and **29** released a lower amount of NO (14.20–14.80 μM) [51].

The vasorelaxant effect of the hybrids was determined by the measurement of the concentration-dependent relaxations of the mesenteric arteries that were preconstructed with 60 mM of potassium chloride (KCl). Cumulative concentration of the NO donors **18a**, **20a**, **26**, and **27** that were applied to the arteries was 10^−10^–10^−3.5^ M and 10^−8^–10^−1^ M for hybrids **17a**, **19a**, **28**, and **29**. Hybrid **26** showed the best activity, with a pIC_50_ value of 6.171, close to that of SNP (pIC_50_ = 6.786). Hybrids **27**, **18a**, and **20a** were not as potent as the control SNP, with pIC_50_ values of 5.875, 5.872, and 5.698, respectively [50]. Phenylfuroxans displayed decreased activity in comparison to SNP (pIC_50_ = 6.786). Hybrids **19a** and **29** were the most potent, with pIC_50_ values of 4.286 and 4.547, respectively, followed by hybrids **17a** (pIC_50_ = 3.778) and **28** (pIC_50_ = 3.538). The vasodilative activities of the target compounds were similar to their antiplatelet aggregation abilities, and also may be positively correlated with their NO-releasing abilities [51].

## 4. Conclusions

The molecular hybridization approach has become a powerful tool in drug design, leading to the creation of novel drug candidates with improved pharmacological properties. In the recent decade, NO-releasing chemical groups were incorporated into the structure of known bioactive molecules to yield hybrid molecules. NO donors are a heterogeneous group of compounds that have the ability to release NO or an NO-related species in vitro or in vivo, independently of its endogenous sources. In addition, cinnamic acid and its derivatives are often used as the main scaffold of new hybrid molecules due to the variety of their biological activities. In this review, many cinnamic acid-NO donor hybrids, mainly nitro-oxy esters and furoxans, were described as new pharmacological agents to treat multifactorial diseases such as inflammation, oxidative stress, atherosclerosis, cancer, Alzheimer’s disease, thrombosis, diabetes, etc. Most of them have shown promising results in in vitro and in vivo assays.

Considering their structural characteristics, hybrids with free phenolic hydroxy groups seem to have better antioxidant, anti-inflammatory, and anticancer activities. The potency decreases when a bulky substitute presents nearby.

Furthermore, the presence within the hybrid structure of the moiety acting as a NO donor is important for the vasodilating activity, antiplatelet aggregation activity, and NO-releasing ability of the novel hybrid. It seems that there is a correlation between these activities in most cases. In addition, the presence of the sulfonyl group may enhance the antiplatelet aggregation activity. Although the research we reviewed did not discuss the role of lipophilicity, further investigation must be conducted to delineate the implication of this significant physicochemical property for the biological activities presented.

In general, the presented review describes all the knowledge published on the cinnamate NO donors, and it will be helpful in the design and further investigation of the synthesis and biological evaluation of cinnamate NO-donor hybrids.

## Figures and Tables

**Table 1:** The in vitro anticancer activities of the hybrid nitrates **9**–**12** in nine human lung cancer cell lines (IC_50_ in μM) [42].

Hybrid	A549	H157	H460	1792	H266	Hop62	1299	292G	Calu1
**9a**	13.2	20.5	29.0	28.5	44.0	19.4	28.3	39.4	>50
**9b**	8.82	12.9	19.5	20.8	24.8	13.1	4.51	28.4	30.1
**9c**	6.39	13.4	12.3	13.0	21.2	12.5	20.8	24.9	26.3
**9d**	37.4	32.1	>50	48.7	>50	>50	>50	>50	>50
**9e**	5.95	4.29	4.98	4.86	9.79	5.13	2.34	21.9	2.48
**10a**	10.8	4.34	3.31	18.1	>50	10.0	8.03	13.5	10.7
**10b**	22.6	5.19	3.52	15.4	23.3	10.0	3.14	16.7	21.7
**10e**	0.40	1.36	2.90	0.41	10.3	4.65	0.41	7.95	0.42
**11a**	15.8	>50	>50	>50	>50	>50	>50	>50	1.42
**11b**	>50	>50	>50	>50	>50	>50	>50	>50	>50
**11c**	>50	>50	>50	>50	>50	>50	>50	>50	>50
**11d**	>50	>50	>50	>50	>50	>50	>50	>50	27.8
**12a**	0.69	5.71	9.58	6.61	>50	10.0	>50	4.98	12.5
**12b**	0.41	>50	>50	>50	>50	>50	>50	>50	>50
**12c**	0.45	>50	>50	>50	>50	>50	>50	>50	>50
**12d**	>50	>50	>50	>50	>50	>50	>50	>50	>50

**Table 2:** The in vitro anticancer activities of hybrid nitrates **9**–**12** in melanoma, cervical, neck and head, and breast cancer cell lines (IC_50_ in μM) [42].

Hybrid	Melanona		Cervical	Neck and Head	Breast
	LOX-IMVI	M14	Hela	M4E	SKBR
**9a**	29.5	42.6	6.70	18.4	17.2
**9b**	20.4	32.5	6.35	20.3	8.92
**9c**	>50	24.5	13.4	21.8	8.06
**9d**	>50	>50	16.3	>50	15.2
**9e**	2.36	3.89	1.00	9.38	6.53
**10a**	18.9	24.8	3.11	20.0	5.77
**10b**	>50	19.9	2.25	17.5	4.55
**10e**	0.43	1.49	0.40	4.31	0.41
**11a**	>50	>50	3.62	>50	>50
**11b**	>50	>50	0.74	>50	>50
**11c**	>50	>50	>50	>50	>50
**11d**	>50	>50	>50	>50	>50
**12a**	6.94	8.96	>50	14.1	4.81
**12b**	>50	>50	3.93	>50	3.23
**12c**	7.23	>50	5.73	>50	2.71
**12d**	>50	>50	1.35	>50	39.7

**Table 3:** The in vitro anticancer activities of the hybrid nitrates **17**–**19a** in nine human lung cancer cell lines (IC_50_ in μM) [42].

Hybrid	A549	H157	H460	1792	H266	Hop62	1299	292G	Calu1
**17a**	2.06	4.53	7.03	11.1	13.4	8.66	12.9	16.5	22.9
**17b**	0.72	24.7	11.6	>50	>50	>50	>50	>50	>50
**17c**	3.39	43.1	15.8	8.15	>50	30.4	28.5	31.0	28.8
**17d**	>50	>50	29.4	>50	>50	>50	>50	>50	>50
**17e**	17.2	>50	44.2	>50	>50	>50	>50	>50	>50
**18a**	>50	>50	>50	>50	>50	>50	>50	>50	>50
**18b**	0.42	0.41	0.42	0.40	0.43	0.42	0.41	1.77	0.45
**18c**	0.40	0.42	0.45	0.42	0.40	0.41	0.42	1.93	0.41
**18d**	0.41	0.44	0.43	1.07	0.42	0.48	0.49	2.88	0.45
**19a**	2.71	>50	2.54	3.78	14.2	6.12	>50	9.17	7.68

**Table 4:** The in vitro anticancer activities of the hybrid nitrates **17**–**19a** in melanoma, cervical, neck and head, and breast cancer cell lines (IC_50_ in μM) [42].

Hybrid	Melanona		Cervical	Neck and Head	Breast
	LOX-IMVI	M14	Hela	M4E	SKBR
**17a**	13.8	14.0	7.82	3.37	4.98
**17b**	49.4	>50	>50	44.6	14.2
**17c**	>50	>50	12.1	>50	11.0
**17d**	>50	>50	5.64	>50	4.18
**17e**	>50	>50	>50	>50	11.7
**18a**	>50	>50	>50	>50	>50
**18b**	0.41	0.41	0.40	1.18	0.40
**18c**	0.44	0.40	0.46	0.84	0.42
**18d**	0.42	0.41	0.43	1.12	0.41
**19a**	13.3	12.5	1.23	12.9	0.70

**Table 5:** The in vitro antitumor activity of compounds **21**–**25** against hepatocellular carcinoma and human breast cancer cells (IC_50_ in μM) [47].

Compound	In Vitro Cytotoxicity		
	SMMC-7721	HepG2	MCF-7
**21**	6.1	7.3	3.8
**22**	7.9	8.2	6.5
**23**	8.9	7.8	5.0
**24**	10.8	7.7	6.3
**25**	20.3	18.8	11.2
Adriamycin	0.9	2.1	1.7

## Abbreviations

NO	Nitric Oxide
eNOS	Endothelial Nitric Oxide Synthase
nNOS	Neuronal Nitric Oxide Synthase
iNOS	Inducible Nitric Oxide Synthase
COX	Cyclooxygenase
LOX	Lipoxygenase
ATP	Adenosine Triphosphate
NSAIDs	Non-Steroidal Anti-Inflammatory Drugs
NDGA	Nordihydroguaiaretic Acid
ROS	Reactive Oxygen Species
LDL	Low-Density Lipoprotein
DPPH	2,2-diphenyl-1-picrylhydrazyl
SNAP	*S*-nitroso-*N*-acetylpenicillamine
PGF2a	Prostaglandin F2alpha
ISDN	Isosorbide Ninitrate
ACh	Acetylcholine
AChE	Acetylcholinesterase
BuChE	Butyrylcholinesterase
NBP	3-*n*-butylphtalide
SFDA	State Food and Drug Administration
PRP	Platelet-Rich Plasma
A-V	Arteriovenous
CVD	Cardiovascular Disease
T2DM	Type 2 Diabetes Mellitus
AGEs	Advanced Glycation End-Products
BSA	Bovine Serum Albumin
MGO	Methylglyoxal
ADP	Adenosine Diphosphate
